# The impact of slope-adjusted visit-to-visit body mass index variability on early dementia risk prediction

**DOI:** 10.1038/s41366-026-02052-z

**Published:** 2026-03-13

**Authors:** Michihiro Satoh, Hiroki Nobayashi, Takahito Yagihashi, Yutaro Iwabe, Seiya Izumi, Takahisa Murakami, Yuya Suzuki, Maya Toyama, Shingo Nakayama, Tomoko Muroya, Juichi Fujimori, Hirohito Metoki

**Affiliations:** 1 Division of Public Health, Hygiene and Epidemiology, Faculty of Medicine, Tohoku Medical and Pharmaceutical University, Sendai, Japan; 2 Department of Preventive Medicine and Epidemiology, Tohoku Medical Megabank Organization, Tohoku University, Sendai, Japan; 3 Department of Pharmacy, Tohoku Medical and Pharmaceutical University Hospital, Sendai, Japan; 4 Division of Nephrology and Hypertension, Department of Internal Medicine, The Jikei University School of Medicine, Tokyo, Japan; 5 Division of Neurology, Faculty of Medicine, Tohoku Medical and Pharmaceutical University, Sendai, Japan; 6 Center for Clinical Research Promotion and Development, Tohoku Medical and Pharmaceutical University Hospital, Sendai, Japan; 7 Department of Obstetrics and Gynecology, Tohoku University Graduate School of Medicine, Sendai, Japan; 8 Division of Aging and Geriatric Dentistry, Department of Rehabilitation Dentistry, Tohoku University Graduate School of Dentistry, Sendai, Japan; 9 Department of Nephrology, Southern Tohoku Research Institute for Neuroscience Southern Tohoku General Hospital, Fukushima, Japan; 10 Department of Nephrology, Self-Defense Forces, Sendai Hospital, Sendai, Japan; 11 Department of Pathology and Laboratory Medicine, Cedars-Sinai Medical Center, Los Angeles, CA USA; 12 Division of Internal Medicine, Izumi Hospital, Sendai, Japan; 13 Nanatsumori Family Clinic, Miyagi, Japan; 14 Tohoku Institute for the Management of Blood Pressure, Sendai, Japan

**Keywords:** Risk factors, Epidemiology

## Abstract

**Background:**

Previous studies have not typically separated body mass index (BMI) slope and variability as distinct constructs when examining dementia risk. This study assessed the association between the slope-adjusted visit-to-visit BMI variability and dementia risk.

**Subjects/methods:**

We conducted a retrospective cohort study using Japanese national health insurance data (2015–2023) of individuals aged 50–74 years who underwent five annual health checkups. BMI variability was assessed using the slope-adjusted standard deviation (SD) to account for underlying temporal trends. The proxy outcome for dementia was antidementia drug initiation, analyzed using Fine-Gray competing risk models, accounting for death as a competing risk.

**Results:**

During the mean 2.17 ± 1.19 years of follow-up among 303,042 participants (mean age: 66.6 years, men: 38.6%), antidementia drugs (predominantly donepezil: 67.4%) were initiated in 665 and 2394 died. After adjusting for covariates including BMI at baseline and annual BMI change, the highest tertile of slope-adjusted BMI-SD (≥0.50 kg/m²) was significantly associated with increased dementia risk compared with the lowest tertile (≤0.31 kg/m²). Annual BMI change showed a U-shaped association with dementia risk, with pronounced elevation in the first tertile (BMI decline ≤−0.31%, hazard ratio: 1.60, 95% confidence interval: 1.32–1.93). In the basic model including baseline covariates except BMI at baseline, there was no significant difference in the C-statistics improvements when BMI at baseline or adding slope-adjusted BMI-SD (+0.0147 vs +0.0146) were added, while the greatest C-statistics improvement was observed when BMI decline ≤−0.31% was added. The association between the highest slope-adjusted BMI-SD tertile and dementia risk was stronger in females than males (*P* for interaction = 0.0039).

**Conclusions:**

Slope-adjusted visit-to-visit BMI variability is independently associated with dementia risk, particularly among females, while BMI decline patterns are strong risk factors of dementia. Incorporating longitudinal monitoring of visit-to-visit BMI variability into routine dementia screening may be beneficial.

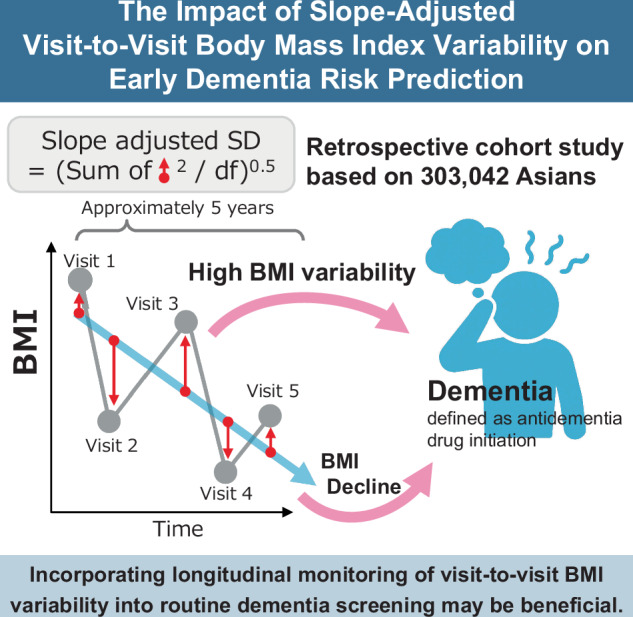

## Introduction

Dementia has rapidly become a global public health concern. The prevalence of dementia has been estimated to increase from 57 million in 2019 to 153 million by 2050 worldwide [1]. To mitigate its societal and economic burden, early prediction through timely behavioral and clinical intervention is crucial.

Body mass index (BMI), particularly low BMI, is associated with a high risk of dementia [2]. Since weight loss has also been reported as a risk factor for dementia [2–11], measuring long-term trends in BMI may be crucial for precise dementia risk prediction. Furthermore, within-person variability in weight or BMI has more recently been linked to cognitive decline and dementia risk [7, 12–17]. Worse glucose tolerance, inflammation, and metabolic factors are considered to be possible mediators contributing to this association [18–22]. However, traditional variability indices that rely on standard deviation (SD), including the coefficient of variation, have computational limitations. These metrics inherently produce higher variability estimates for individuals with steeper slopes, even if the trends are stable. This results in artificially elevated variability measures in individuals with pronounced slopes. The variability indices should preferably be adjusted by the corresponding slope to rigorously explore the effect of variability on differential risk. Despite this problem, previous studies have not considered slope in their analysis of variability measures [7, 12–17]. Another issue in these studies could be the number of measurement points because they mainly included individuals who underwent measurement only three times [7, 12–17]. With such a small number of observations, the calculated variability may largely reflect measurement error or random fluctuations rather than true biological variation, raising questions regarding the validity of these variability estimates.

Given these considerations, the present study aimed to assess the association between slope-adjusted visit-to-visit variability in BMI and the risk of dementia using annually measured data recorded at the five time points from Asian health check-up and claims data. By examining the predictive ability of other indices, including the latest BMI values and slopes, we explored whether measuring visit-to-visit BMI variability provided additional practical benefits for dementia prediction.

## Methods

### Study design and population

This retrospective cohort study used the data from 2015 to 2023 from insurer-based databases provided by DeSC Healthcare Inc. (Tokyo, Japan). The DeSC National Health Insurance (NHI) database includes health checkups and claims data from self-employed individuals, unemployed individuals, or farmers aged <75 years [23–25]. When individuals reach the age of 75 years, all insured individuals are forced to switch their insurance to the “Later-Stage Elderly Healthcare System” in Japan; the follow-up is discontinued at this point due to the change in insurance identification number. The claims database includes data on all inpatient, outpatient, and pharmacy claims received by the insurers. This study was approved by the Research Ethics Committee for Life Science and Medical Research of Tohoku Medical and Pharmaceutical University, and the requirement for informed consent was waived because all data were fully de-identified before analysis (approval ID: 2024-2-015) [26]. The study followed the Strengthening the Reporting of Observational Studies in Epidemiology (STROBE) guidelines [27] and were performed in accordance with the Declaration of Helsinki.

The study design is outlined in Supplementary Fig. S1. We used five annual health checkups (Visits 1–5) to define BMI variability. Visit 5, i.e., the last checkup, was defined as the baseline.

### Participant selection

**Fig. 1:**
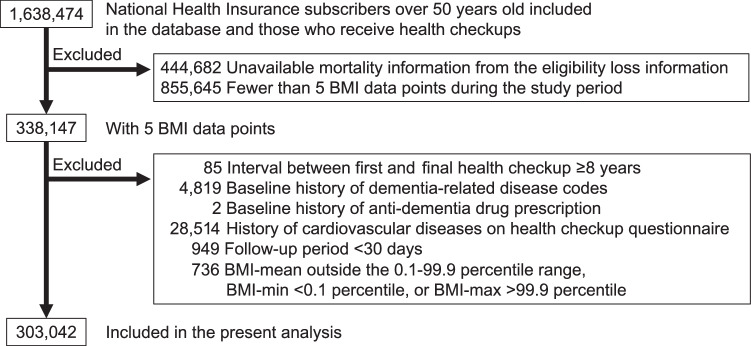
Study population selection flowchart. BMI body mass index.

The participant selection process is illustrated in Fig. 1. Data were available for 1,638,474 individuals. To consider the outcome of death as a competing risk, 444,682 patients whose death information was not available were excluded. Additionally, 855,645 individuals without data from the five available annual health checkups to define visit-to-visit BMI variability were excluded. Of the remaining 338,147 individuals, individuals with intervals longer than eight years between the initial and final health examinations, those with a history of dementia-related diagnostic codes, those with a history of antidementia medication prescription, those with a history of cardiovascular diseases (from the health examination questionnaire) at baseline, and those with a follow-up period of less than 30 days were further excluded. To eliminate the effects of outliers, individuals with extreme BMI values outside the 0.1–99.9 percentile range were excluded. Finally, data from 303,042 individuals were analyzed. This observational study included all available eligible participants; no a priori sample size calculation was performed.

### Baseline data

All health checkups in Japan are encouraged to be performed under the guidelines of the Japanese Ministry of Health, Labour, and Welfare [28]. Body mass index was calculated as weight in kilograms divided by height in meters squared (kg/m²). Blood and urine samples were collected at the same time as the other examination. Blood pressure (BP), hemoglobin A1c (HbA1c) level, low-density lipoprotein cholesterol (LDL) level, and proteinuria by dipstick test, which are commonly measured for detecting metabolic syndrome in Japan, were used as covariates. Information on smoking status, alcohol consumption, and history of cardiovascular disease were collected using a questionnaire at each health checkup.

The use of lipid-lowering (World Health Organization Anatomical Therapeutic Chemical [WHO-ATC] code: C10), antidiabetic (ATC code: A10), and antihypertensive medications (ATC code: C02CA, C02A, C03AA, C03BA, C03CA01, C03DA, C03DB, C08CA, C08DB, C09, C07A, C02DB, G04CA03) was confirmed using claims data from within 365 days before the baseline (Detailed codes are shown elsewhere [29, 30]).

### Visit-to-visit BMI variability indices

As the primary measure of visit-to-visit BMI variability, the slope-adjusted BMI-SD was calculated as the SD of residuals derived from individual linear regression analyses, eliminating the influence of underlying temporal trends from the variability estimates (Supplementary Fig. S2). This approach addresses the computational limitation in which traditional SD-based metrics artificially inflate the variability in individuals with steeper BMI trajectories. Additional variability indices included the coefficient of variation (CV), average real variability (ARV), and the maximum minus minimum difference (MMD). Visit-to-visit percentage changes were calculated as the percentage difference between consecutive measurement points (i.e., the relative change from one visit to the next immediate visit). As CV values are influenced by both positive and negative slope values, the absolute values of the annual BMI change (|BMI annual change|) were used for the slope analyses.

The variability indices had positively skewed distributions, and thus were naturally log (ln) transformed. To account for potential measurement errors and extreme outliers in the BMI variability indices, slope-adjusted BMI-SD, CV, ARV, and MMD values below the 0.1st percentile and above the 99.9th percentile were replaced with the 0.1st and 99.9th percentile values, respectively, in a process called winsorization.

### Outcome definitions

The initial prescription of the following antidementia drugs (WHO-ATC code) was defined as a proxy for incident dementia: donepezil (N06DA02), rivastigmine (N06DA03), galantamine (N06DA04), ipidacrine (N06DA05), and memantine (N06DX01). We did not use the initial definitive diagnosis of dementia (ICD-10 codes F00–03 and G30–G31 [with the exception of G319]) recorded in the claims data because its reliability remains uncertain; the diagnosis documented in the claims data could be assigned so that various clinical examinations would be approved by insurance [23].

Death outcome was extracted from the information on loss of eligibility as an NHI-insured person and was used as a competing risk. Censoring was defined as the end of the database period or loss of eligibility for reasons other than death.

### Statistical analysis

Differences among groups were compared by assessing standardized mean differences (SMDs) [31]. An SMD of |0.1| was set as the cutoff point to determine the group difference [31]. Unless otherwise specified, continuous variables are summarized as mean ± SD and categorical variables are expressed as percentages. The correlations between BMI variability indices and annual BMI changes were assessed to confirm the associations between the variability indices and annual changes.

The association between BMI indices and dementia risk was analyzed using Fine-Gray hazard models to account for the competing risk of death [32]. To consider the potential non-linear associations, the participants were categorized into tertiles or sextiles of annual BMI change or slope-adjusted BMI-SD. The group with the lowest risk was used as the reference. The hazard ratios were calculated using the model with both annual BMI change and slope-adjusted BMI-SD categories and were adjusted for sex and baseline characteristics, including age, smoking status, alcohol consumption, proteinuria, systolic BP, HbA1c, LDL, and the use of antihypertensive, antidiabetic, or lipid-lowering drugs, as identified from the claims data. Missing continuous variables for systolic BP (*n* = 5, 0.002%), diastolic BP (*n* = 6, 0.002%), HbA1c levels (*n* = 2145, 0.71%), LDL levels (*n* = 152, 0.05%), and high-density lipoprotein levels (*n* = 9, 0.003%) were interpolated from the regression slope of age after sex stratification. For participants with an unknown smoking (*n* = 9, 0.003%), alcohol consumption (*n* = 13,194, 4.35%), and proteinuria status (*n* = 441, 0.15%), the design variable was imputed to the sex-specific mean of the codes (0, 1).

Model fit was assessed using the Akaike Information Criterion (AIC), with lower values indicating better model fit [33]. The discriminative ability of each model was evaluated using the C-statistic calculated at two years, based on the time-dependent area under the receiver operating characteristic curve (AUC) for competing risk analysis. Confidence intervals for C-statistics were obtained using the DeLong method [34] as implemented in the pec package’s Score function. To evaluate the incremental predictive value of adding variables to the models, the AUC differences between the models were calculated using their corresponding 95% confidence intervals (CIs).

For sensitivity analyses, we examined alternative BMI variability indices other than slope-adjusted BMI-SD. In addition, visit-to-visit differences between adjacent time points were evaluated to determine their association with the risk of dementia. In addition, stratified analyses according to these baseline covariates as well as subgroup analyses based on a follow-up duration of two years (median) and median annual BMI change were performed. Interaction was tested using an interaction variable calculated as a stratification factor multiplied by the primary independent variable.

To calculate C-statistics and AIC with the cmprsk package for competing risk analysis, riskRegression package for model evaluation, and pec package for performance assessment R (Ver 4.4.1) was used. The Fine-Gray models were fitted using the FGR function from the riskRegression package. All other analyses were conducted using the SAS software (version 9.4 1M8; SAS Institute Inc., Cary, NC, USA). Statistical significance was set at α < 0.05, and all tests were two-sided. Continuous variables are presented as means ± standard deviations unless otherwise noted.

## Results

### Baseline characteristics

The baseline characteristics of the participants stratified by slope-adjusted BMI-SD tertiles are shown in Table 1. Compared with the lowest BMI variability tertile, participants in the highest tertile were younger, more likely to be prescribed antidiabetic or antihypertensive drugs, and had higher HbA1c levels (|SMD|≥0.1). As expected, BMI variability indices and BMI-related measures increased substantially across tertiles, with participants in higher tertiles having progressively higher mean BMI values and greater annual changes in absolute BMI.

**Table 1:** Characteristics by slope-adjusted BMI-SD.

	Tertiles of slope-adjusted BMI-SD, kg/m^2^			
Characteristics	Tertile 1: ≤0.31	Tertile 2: 0.31–0.50	Tertile 3: ≥0.50	SMD T1 vs T3
*N*	101,012	101,017	101,013	
Age, years	66.8 ± 5.2	66.7 ± 5.4	66.2 ± 5.8	-0.12
Male, %	36.7	39.4	39.7	0.06
Smoker, %	9.3	10.5	11.3	0.07
Alcohol consumption, %	23.1	23.2	19.9	-0.08
Urine protein, %	3.7	4.2	5.2	0.07
Systolic BP, mmHg	129.3 ± 17.3	129.8 ± 17.1	130.1 ± 17.1	0.05
Diastolic BP, mmHg	75.7 ± 11.1	75.8 ± 10.7	76.0 ± 11.5	0.03
HbA1c, %	5.7 ± 0.5	5.8 ± 0.6	5.8 ± 0.7	0.12
LDL cholesterol, mg/dL	124.9 ± 33.5	124.3 ± 29.6	123.2 ± 30.5	-0.05
Antihypertensive treatment, %	34.3	37.2	40.8	0.13
Antidiabetic treatment, %	7.1	8.5	12.2	0.17
Lipid-lowering treatment, %	32.8	33.6	35.6	0.06
Mean BMI, kg/m²	22.1 ± 3.0	22.7 ± 3.2	23.7 ± 3.5	0.48
BMI 1st, kg/m²	22.0 ± 3.0	22.6 ± 3.1	23.7 ± 3.6	0.51
BMI 2nd, kg/m²	22.1 ± 3.0	22.7 ± 3.2	23.7 ± 3.6	0.48
BMI 3rd, kg/m²	22.1 ± 3.0	22.7 ± 3.2	23.7 ± 3.6	0.47
BMI 4th, kg/m²	22.2 ± 3.1	22.8 ± 3.2	23.7 ± 3.6	0.46
BMI 5th (baseline), kg/m²	22.2 ± 3.1	22.8 ± 3.3	23.7 ± 3.7	0.43
Annual BMI change, %	0.2 ± 1.0	0.2 ± 1.1	0.0 ± 1.6	-0.13
Absolute value of annual BMI change, %	0.60 (0.28–1.07)	0.70 (0.32–1.23)	0.93 (0.43–1.67)	0.50
BMI-CV, %	1.35 (1.00–1.97)	2.00 (1.63–2.60)	3.26 (2.58–4.37)	1.47
BMI-ARV, kg/m²	0.29 (0.22–0.36)	0.48 (0.41–0.57)	0.81 (0.66–1.02)	2.09
BMI-MMD, kg/m²	0.73 (0.54–1.06)	1.10 (0.91–1.43)	1.88 (1.48–2.51)	1.56
Slope-adjusted BMI-SD, kg/m²	0.22 (0.16–0.26)	0.39 (0.35–0.44)	0.68 (0.58–0.87)	2.29

Due to their non-normal distribution, the BMI variability indices were expressed as the median (interquartile range).

*BP* blood pressure, *CV* coefficient of variation, *ARV* average real variability, *MMD* min-max difference, *SD* standard deviation, *LDL* low-density lipoprotein, *BMI* body mass index.

The absolute value of annual BMI change was moderately to strongly correlated with ln BMI-CV, ln BMI-ARV, and ln BMI-MMD (*r* ≥ 0.43). The slope-adjusted BMI-SD exhibited the weakest correlation with the absolute value of annual BMI change (r = 0.25). Slope-adjusted BMI-SD strongly correlated with other variability indices (*r* ≥ 0.73) (Supplementary Table S1).

### BMI variability or annual change and dementia risk

During a mean follow-up of 2.17 ± 1.19 (median 2.15 [interquartile range: 1.13–3.39]) years, antidementia drugs were initiated in 665 participants and 2394 died. Donepezil (N06DA02) was prescribed to 448 patients (67.4%), followed by galantamine (N06DA04) to 90 (13.5%), memantine (N06DX01) to 79 (11.9%), and rivastigmine (N06DA03) to 48 (7.2%).

**Fig. 2:**
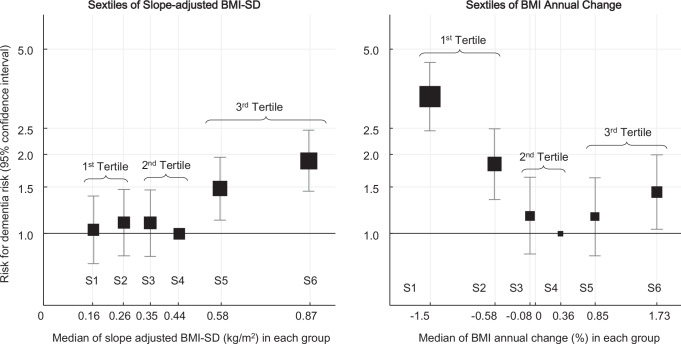
Association between BMI trajectories and dementia risk. Hazard ratios for antidementia drug initiation across sextiles for annual BMI change from baseline (left panel) and slope-adjusted BMI-SD (right panel). Both BMI trends and variability measures were simultaneously included in the models with covariates to assess independent associations. The models were adjusted for age, sex, smoking status, alcohol consumption, proteinuria, systolic blood pressure, HbA1c and LDL cholesterol levels, medication use, and baseline BMI. The lowest risk group was used as the reference category. BMI body mass index, HbA1c hemoglobin A1c, LDL low-density lipoprotein, SD standard deviation.

Figure 2 shows the dementia risk according to the slope-adjusted BMI-SD and sextiles of annual BMI changes, which were included in the same model. Dementia risk did not differ across the 1st to 4th sextiles of slope-adjusted BMI-SD, whereas it was higher in the 5th and 6th sextile groups, i.e., the highest tertile group of slope-adjusted BMI-SD. The association between the annual BMI changes and dementia risk was U-shaped.

Table 2 lists the incremental predictive values of the different BMI-related measures. When the model with baseline characteristics but without BMI (Model 1) was treated as the reference model, annual BMI change ≤−0.31% revealed the best predictive ability with the lowest AIC (Model 4), followed by BMI at baseline (Model 2), slope-adjusted BMI-SD (Model 6), mean BMI (Model 3), and annual BMI change ≥1.19% (6th sextile) (Model 5); there was no significant difference in the C-statistic improvements when BMI at baseline and slope-adjusted BMI-SD were added to Model 1 (Model 2 vs Model 6, −0.0001 [95% CI: −0.0007 to 0.0004]). Further addition of slope-adjusted BMI-SD to the model with baseline BMI and annual BMI change categories provided significant incremental improvements (Model 9).

**Table 2:** Model performance comparison for dementia risk prediction at two years.

Model	Variables included	AIC	C-statistics (95% CI)	Compared reference model	Improvement (95% CI)
Model 1	Age, Sex, Smoking status, Alcohol consumption, Proteinuria, SBP, HbA1c, LDL, and the use of antihypertensive/antidiabetic/lipid-lowering drugs at baseline	15,568	0.741 (0.741–0.742)	–	–
Model 2	Model 1 + BMI at baseline	15,499	0.756 (0.756–0.756)	Model 1	+0.0147 (0.0141–0.0153)
Model 3	Model 1 + Mean BMI	15,532	0.750 (0.749–0.750)	Model 1	+0.0084 (0.0078–0.0090)
Model 4	Model 1 + Annual BMI change ≤−0.31% (1st and 2nd sextiles^a^)	15,426	0.768 (0.768–0.769)	Model 1	+0.0269 (0.0263–0.0275)
Model 5	Model 1 + Annual BMI change ≥1.19% (6th sextile^a^)	15,565	0.744 (0.744–0.744)	Model 1	+0.0025 (0.0020–0.0031)
Model 6	Model 1 + Slope-adjusted BMI-SD ≥ 0.5 kg/m^2^ (5th and 6th sextiles^a^)	15,522	0.756 (0.756–0.756)	Model 1	+0.0146 (0.0140–0.0151)
Model 7	Model 6 + Annual BMI change ≤−0.31% (1st and 2nd sextiles^a^)	15,391	0.774 (0.773–0.774)	Model 2	+0.0176 (0.0170–0.0182)
Model 8	Model 7 + Annual BMI change ≥1.19% (6th sextile^a^)	15,387	0.775 (0.774–0.775)	Model 7	+0.0009 (0.0004–0.0015)
Model 9	Model 8 + Slope-adjusted BMI-SD ≥ 0.5 kg/m^2^ (5th and 6th sextiles^a^)	15,346	0.784 (0.784–0.784)	Model 8	+0.0094 (0.0088–0.0099)

C-statistics were calculated at two years using the time-dependent area under the receiver operating characteristic curve for competing risk analysis.

^a^The 1st–2nd sextile [1st tertile] and 6th sextile of annual BMI change and the 5th–6th sextile [3rd tertile] of slope-adjusted BMI-SD were significantly associated with dementia risk as show in Fig. 2.

*AIC* Akaike Information Criterion, *BMI* body mass index, *CI* confidence interval, *HbA1c* hemoglobin A1c, *LDL* low-density lipoprotein, *SBP* systolic blood pressure, *SD* standard deviation.

### Sensitivity analyses

Similar patterns were observed across all BMI variability indices, with the highest tertile consistently showing an increased risk compared to the reference groups (Supplementary Table S2). The associations between visit-to-visit differences from Visits 1 to 2, 2 to 3, 3 to 4, and 4 to 5 and dementia risk were not consistent (Supplementary Table S2).

**Fig. 3:**
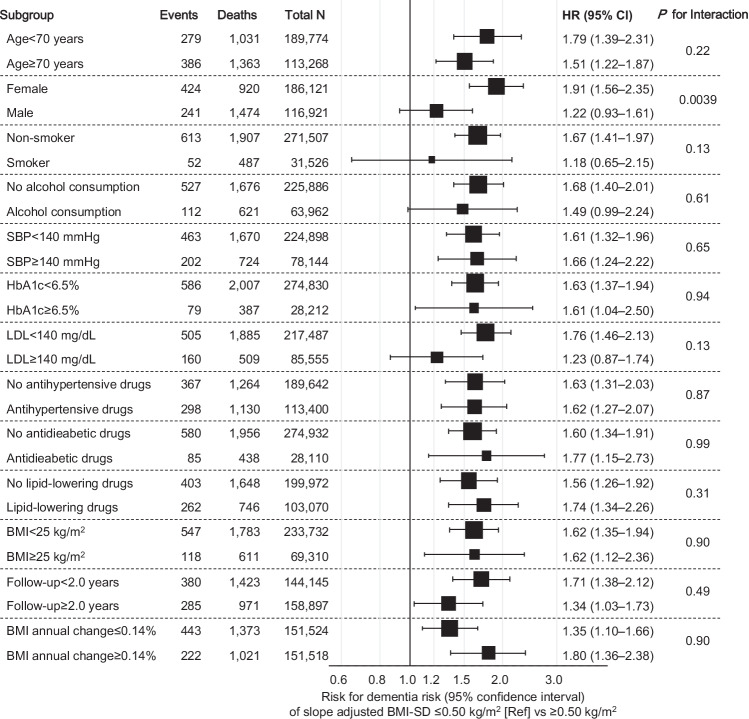
Subgroup analysis of BMI variability and dementia risk. Hazard ratios for antidementia drug initiation comparing low-to-moderate (tertiles 1–2: ≤0.50 kg/m²) vs high slope-adjusted BMI-SD (tertile 3: ≥0.50 kg/m²) across subgroups. The square marker size is proportional to the number of events. All models were adjusted for age, sex, smoking status, alcohol consumption, proteinuria, systolic blood pressure, HbA1c and LDL levels, medication use, and BMI category. BMI body mass index, LDL low-density lipoprotein, SBP systolic blood pressure, HbA1c hemoglobin A1c, SD standard deviation.

The association between the highest slope-adjusted BMI-SD tertile and dementia risk was assessed after stratification according to age (<70/≥70 years), sex, smoking status, alcohol consumption, BP levels (<140/≥140 mmHg), HbA1c levels (<6.5%/≥6.5%), LDL levels (<140/≥140 mg/dL), medication use (antihypertensive, antidiabetic, and lipid-lowering drugs), baseline BMI (<25/≥25 kg/m²), follow-up duration (<2.0/≥2.0 years), and annual BMI change (≤0.14%/≥0.14%). Among these subgroup analyses, a significant interaction was observed only for sex (*P* for interaction = 0.0039) (Fig. 3). After stratification by sex, the association between slope-adjusted BMI-SD and dementia risk was clearer in females (Supplementary Fig. S3) than in males (Supplementary Fig. S4). Improvements in C-statistics when slope-adjusted BMI-SD was added were observed in females (Supplementary Table S3) but not in males (Supplementary Table S4).

## Discussion

This large-scale longitudinal study demonstrated that slope-adjusted BMI-SD is independently associated with an increased risk of dementia, which was assessed considering antidementia drug initiation as the proxy outcome. The predictive value of the slope-adjusted BMI-SD for dementia remained significant after adjusting for annual BMI changes. Slope-adjusted BMI-SD provided additional prognostic information, with more pronounced associations in females than males. Meanwhile, annual BMI decline by ≤−0.31% was consistently associated with the risk of dementia regardless of sex and was the strongest predictor of dementia among the BMI-related indices.

The slope-adjusted BMI-SD significantly predicted future dementia risk. Only the third tertile exhibited a significantly elevated dementia risk, implying a nonlinear association between BMI variability and dementia risk. This index also achieved a C-statistic improvement comparable to that of the baseline BMI, suggesting its potential value for long-term dementia risk assessment if sufficient longitudinal data are available.

The present study focused specifically on evaluating whether slope-independent variability predicts dementia risk, rather than comprehensively comparing all available BMI variability indices. Although conventional variability indices such as BMI-CV, BMI-MMD, and BMI-ARV were also significantly associated with a higher risk of dementia, they were highly correlated with absolute annual BMI change, indicating potential confounding. In contrast, slope-adjusted BMI-SD may offer a more valid and independent measure of intra-individual BMI variability, minimizing the influence of secular trends such as annual BMI change. Notably, BMI changes between consecutive visits (e.g., change in BMI from Visit 4 to Visit 5) showed inconsistent associations and did not improve risk prediction, highlighting the importance of multiple repeated measurements over time to capture meaningful variability. The statistically significant improvement suggests slope-adjusted variability captures complementary information independent of directional weight change. However, the improvement in C-statistics achieved by adding slope-adjusted BMI-SD to the model accounting for baseline BMI, BMI change, and all other risk factors was modest (+0.0094). Future studies employing more sophisticated analytical approaches, including machine learning algorithms and decision curve analysis, are warranted to determine the optimal BMI variability index for dementia prediction.

Although the direct mechanisms underlying our findings remain unclear, repeated weight cycling in obese mice has been reported to cause glucose intolerance [18]. Brain insulin resistance can occur in patients with Alzheimer’s disease [19], which may be caused by weight variability. Weight fluctuations are closely linked to central nervous system regulation via hormonal signals such as leptin, insulin, and gut-derived peptides [22], and impairment of this complex regulatory system may mediate our findings. Furthermore, body weight variability has been associated with inflammation and cardiovascular risk [20, 21, 35, 36], which may also contribute to dementia risk through vascular mechanisms. Meanwhile, the association between slope-adjusted BMI-SD and dementia risk was significant only in females but not in males. Although the exact reasons for the sex-specific discrepancies remain uncertain, females experience steep estrogen decline during menopause, losing estrogen’s protective effects against amyloid β-protein accumulation suddenly [37–39]. Additionally, the APOE ε4 allele confers disproportionately greater dementia risk in females compared to males [40, 41], and genetic polymorphisms including APOE variants influence both Alzheimer’s disease susceptibility and BMI regulation [42]. This convergence of hormonal changes, genetic predisposition, and metabolic dysregulation may explain why BMI variability serves as a particularly sensitive marker of dementia risk in females. Another possible reason for sex differences may be lifestyle factors. In Japan, women, particularly the older generations, have traditionally been primarily responsible for preparing household meals. Therefore, cognitive decline in women may directly influence BMI variability through changes in dietary patterns and food preparation. However, sex differences in the association between body weight variability and the risk of dementia remain controversial. Some studies reported stronger associations in females [7, 16, 43], while others found stronger associations in males [14, 17] and several studies reported no significant sex interactions [11, 15]. Further studies are needed to clarify this issue.

Despite the significant predictive value of the slope-adjusted BMI-SD, the AIC values and C-statistics improvement analysis revealed that BMI decline by ≤−0.31% annually demonstrated the greatest improvement in predictive performance among the BMI related indices. This improvement achieved by adding the annual BMI decline to the prediction model exceeded that achieved by adding the baseline BMI or mean BMI, highlighting the critical importance of detecting BMI decline patterns in dementia risk assessment. In longitudinal studies with over 20 years of follow-up, BMI decline demonstrated an onset approximately 10 years prior to dementia diagnosis and exhibited a linear decrease during the past 5 years [9, 10]. In contrast, the contribution of annual BMI increase to model improvement was limited. This could be because the definition of antidementia drug initiation in the present study strongly reflects the incidence of nonvascular diseases. A previous meta-analysis revealed that ≥0.5% weight gain per year tended to be associated with vascular dementia but not with non-vascular dementia [2]. A recent study demonstrated that weight loss or gain during 1 year was significantly associated with the incidence of functional disability and all-cause mortality in older adults [44]. Therefore, weight change is not a negligible factor considered when assessing future general health status in older adults.

This study has several limitations. First, our outcome definition was based on antidementia drug initiation, which may have overlooked patients who received non-pharmacological interventions for cognitive impairment. This precludes accurate estimation of dementia incidence rates or risk differences and reduces statistical power. However, antidementia drugs are rarely prescribed for individuals without cognitive impairment, suggesting the high specificity of our definition, leading to accurate relative risk estimation [23]. Second, we were unable to examine actual dementia subtypes separately, such as Alzheimer’s disease, dementia with Lewy bodies, or other specific forms of dementia. Furthermore, data on the cognitive scales used to evaluate the severity or progression of cognitive decline were lacking. Third, the study population was restricted to participants who attended all five annual health checkups, which likely introduced a healthy selection bias and may limit the generalizability of our findings to a broader population. Fourth, the relatively short mean observation period of two years may not have captured the full spectrum of cognitive decline. Given that the progression from subjective cognitive impairment to amnestic mild cognitive impairment can span approximately a decade [45], the observed BMI variability may have been influenced by an underlying cognitive impairment that had not yet reached the threshold for antidementia drug prescription. Finally, other confounding factors such as education level and economic status that could not be captured in the present database may have influenced our findings.

In conclusion, high BMI variability, indicated by slope-adjusted BMI-SD, provided additional prognostic information, with associations that were particularly pronounced in females. Use of the slope-adjusted variability index enhanced the plausibility of the observed association between long-term visit-to-visit BMI variability and dementia risk. Meanwhile, annual BMI decline emerged as a strong predictor, regardless of sex, outperforming baseline BMI measures in predictive performance. These findings support the integration of not only a single BMI point but also longitudinal BMI monitoring into routine dementia risk assessment strategies. Future research with long-term follow-up is needed to establish causality and to explore optimal intervention strategies for individuals with different BMI patterns.

## Supplementary information

Supplemental Material

## Data Availability

The data in this study are not authorized for use by third parties under a contract with DeSC Healthcare, Inc. The authors can provide additional analyses upon request. The DeSC database is available to anyone who purchases it from DeSC Healthcare Inc.

## Code availability

Available from the first author upon request.
