# Supplementary material for: The impact of slope-adjusted visit-to-visit body mass index variability on early dementia risk prediction
Source: Int J Obes (Lond). 2026 Mar 13;50(5):1115–23. doi: 10.1038/s41366-026-02052-z (PMC13226059; doi:10.1038/s41366-026-02052-z)
Supplement: Supplementary file 1 — Supplemental Material [file 41366_2026_2052_MOESM1_ESM.pdf]

Supplementary Materials

The authors have provided this online data supplement to readers with additional information regarding this study.

Supplement to:

The Impact of Slope-Adjusted Visit-to-Visit Body Mass Index Variability on Early Dementia Risk Prediction

Correspondence:
Michihiro Satoh, PhD,
Assistant Professor (Lecturer), Division of Public Health, Hygiene and Epidemiology, Faculty of Medicine, Tohoku Medical and Pharmaceutical University, 1-15-1 Fukumuro, Miyagino-ku, Sendai, Miyagi 983-8536, JAPAN
E-mail: satoh.mchr@tohoku-mpu.ac.jp
Official Twitter account: @TohokuMPU_PHHE
TEL: +81-22-290-8727; FAX: +81-22-290-8728

Table S1. Pearson Correlation Coefficients Among BMI-Related Variables

	ln Slope-adjusted BMI-SD	ln BMI-CV	ln BMI-ARV	ln BMI-MMD	Mean BMI	Annual BMI change
ln Slope-adjusted BMI-SD	—	—	—	—	—	—
ln BMI-CV	0.725†	—	—	—	—	—
ln BMI-ARV	0.902†	0.839†	—	—	—	—
ln BMI-MMD	0.755†	0.961†	0.880†	—	—	—
Mean BMI	0.212†	0.001	0.243†	0.243†	—	—
Annual BMI change	-0.071†	0.012†	-0.014†	0.029†	0.062†	—
Absolute value of Annual BMI change	0.249†	0.739†	0.430†	0.706†	0.015†	0.108†

All indices, including the mean BMI, were derived from data obtained over five visits.
**P*<0.05, †*P*<0.001
CV, coefficient of variation; ARV, average real variability; MMD, min-max difference; SD, standard deviation; BMI, body mass index; ln, natural log-transformed.

Table S2. Association Between BMI Variability and Anti-dementia Drug Initiation: Hazard Ratios by Tertile Groups

	Event n	Death n	Total n	Hazard ratio (95%CI)
Slope-adjusted BMI-SD, kg/m^2				
Tertile 1: ≤0.31	189	614	101,012	1.02 (0.83–1.25)
Tertile 2: 0.31–0.50	183	741	101,017	1.00 (Reference)
Tertile 3: ≥0.50	293	1,039	101,013	1.60 (1.32–1.93)
BMI-CV, %				
Tertile 1: ≤1.73	151	628	101,012	1.00 (Reference)
Tertile 2: 1.73–2.75	181	691	101,017	1.11 (0.87–1.40)
Tertile 3: ≥2.75	333	1,075	101,013	1.88 (1.45–2.44)
BMI-ARV, kg/m^2				
Tertile 1: ≤0.39	172	603	101,012	1.00 (Reference)
Tertile 2: 0.39–0.62	204	713	101,017	1.16 (0.94–1.42)
Tertile 3: ≥0.62	289	1,078	101,013	1.67 (1.36–2.04)
BMI-MMD, kg/m^2				
Tertile 1: ≤0.95	157	613	101,012	1.00 (Reference)
Tertile 2: 0.95–1.54	192	706	101,017	1.19 (0.95–1.50)
Tertile 3: ≥1.54	316	1,075	101,013	1.97 (1.54–2.52)
(Visit 2-Visit 1)/Visit 1, %				
Tertile 1: ≤-1.88	264	884	101,012	1.12 (0.92–1.37)
Tertile 2: -1.88 – 2.67	221	713	101,017	1.11 (0.91–1.35)
Tertile 3: ≥2.67	180	797	101,013	1.00 (Reference)
(Visit 3-Visit 2)/Visit 2, %				
Tertile 1: ≤-1.73	265	907	101,012	1.06 (0.87–1.28)
Tertile 2: -1.73 – 2.76	193	717	101,017	1.00 (Reference)
Tertile 3: ≥2.76	207	770	101,013	1.20 (0.98–1.46)
(Visit 4-Visit 3)/Visit 3, %				
Tertile 1: ≤-1.87	290	916	101,012	1.19 (0.98–1.44)
Tertile 2: -1.87 – 2.61	187	675	101,017	1.00 (Reference)
Tertile 3: ≥2.61	188	803	101,013	1.11 (0.90–1.37)
(Visit 5-Visit 4)/Visit 4, %				
Tertile 1: ≤-2.12	291	1,006	101,012	1.35 (1.12–1.64)
Tertile 2: -2.12 – 2.36	174	670	101,017	1.00 (Reference)
Tertile 3: ≥2.36	200	718	101,013	1.21 (0.99–1.49)

The lowest risk group was used as the reference category. The results were adjusted for age, sex, smoking status, alcohol consumption, proteinuria, systolic blood pressure, HbA1c levels, LDL levels, medication use, BMI, and sextiles of the annual BMI change. Owing to rounding, some cutoff values appeared in both T1 and T2 or T2 and T3.

BMI, body mass index; HbA1c, hemoglobin A1c; LDL, low-density lipoprotein; CI, confidence interval; CV, coefficient of variation; ARV, average real variability; MMD, min-max difference, SD, standard deviation.

Table S3. Model Performance Comparison for Dementia Risk Prediction at Two Years in Females (n=186,121)

Model	Variables Included	AIC	C-statistics (95% CI)	Compared reference model	Improvement (95% CI)
Model 1	Age, Smoking status, Alcohol consumption, Proteinuria, SBP, HbA1c, LDL, and the use of antihypertensive/antidiabetic/lipid-lowering drugs at baseline	9494	0.758 (0.758–0.758)	-	-
Model 2	Model 1 + BMI at baseline	9446	0.773 (0.773–0.773)	Model 1	+0.0152 (0.0149–0.0155)
Model 3	Model 1 + Mean BMI	9473	0.764 (0.763–0.764)	Model 1	+0.0058 (0.0055–0.0062)
Model 4	Model 1 + Annual BMI change ≤-0.31% (1st and 2nd sextiles*)	9392	0.784 (0.784–0.784)	Model 1	+0.0263 (0.0260–0.0266)
Model 5	Model 1 + Annual BMI change ≥1.19% (6th sextile*)	9489	0.763 (0.763–0.763)	Model 1	+0.0049 (0.0046–0.0053)
Model 6	Model 1 + Slope-adjusted BMI-SD ≥0.5 kg/m^2 (5th and 6th sextiles*)	9444	0.783 (0.783–0.783)	Model 1	+0.0254 (0.0251–0.0257)
Model 7	Model 6 + Annual BMI change ≤-0.31% (1st and 2nd sextiles*)	9368	0.792 (0.791–0.792)	Model 2	+0.0186 (0.0183–0.0189)
Model 8	Model 7 + Annual BMI change ≥1.19% (6th sextile*)	9367	0.791 (0.791–0.791)	Model 7	-0.0002 (-0.0005–0.0001)
Model 9	Model 8 + Slope-adjusted BMI-SD ≥0.5 kg/m^2 (5th and 6th sextiles*)	9321	0.808 (0.808–0.809)	Model 8	+0.0170 (0.0167–0.0173)

C-statistics were calculated at two years using time-dependent areas under the receiver operating characteristic curve for competing risk analysis, with standard errors obtained using the DeLong method.

* The 1st–2nd sextile [1st tertile] and 6th sextile of annual BMI change and the 5th–6th sextile [3rd tertile] of slope-adjusted BMI-SD were significantly associated with dementia risk in as shown in **Figure 2**.

AIC, Akaike Information Criterion; BMI, body mass index; CI, confidence interval; HbA1c, hemoglobin A1c; LDL, low-density lipoprotein; SD, standard deviation.

Table S4. Model Performance Comparison for Dementia Risk Prediction at Two Years in Males (n= 116,921)

Model	Variables Included	AIC	C-statistics (95% CI)	Compared reference model	Improvement (95% CI)
Model 1	Age, Smoking status, Alcohol consumption, Proteinuria, SBP, HbA1c, LDL, and the use of antihypertensive/antidiabetic/lipid-lowering drugs at baseline	5206	0.727 (0.725–0.729)	-	-
Model 2	Model 1 + BMI at baseline	5187	0.736 (0.733–0.738)	Model 1	+0.0086 (0.0055–0.0117)
Model 3	Model 1 + Mean BMI	5192	0.736 (0.733–0.738)	Model 1	+0.0089 (0.0058–0.0120)
Model 4	Model 1 + Annual BMI change ≤-0.31% (1st and 2nd sextiles*)	5167	0.752 (0.749–0.754)	Model 1	+0.0246 (0.0216–0.0276)
Model 5	Model 1 + Annual BMI change ≥1.19% (6th sextile*)	5208	0.727 (0.725–0.729)	Model 1	-0.0000 (-0.0031–0.0031)
Model 6	Model 1 + Slope-adjusted BMI-SD ≥0.5 kg/m^2 (5th and 6th sextiles*)	5204	0.726 (0.724–0.728)	Model 1	-0.0011 (-0.0042–0.0020)
Model 7	Model 6 + Annual BMI change ≤-0.31% (1st and 2nd sextiles*)	5158	0.751 (0.749–0.753)	Model 2	+0.0157 (0.0126–0.0188)
Model 8	Model 7 + Annual BMI change ≥1.19% (6th sextile*)	5156	0.755 (0.753–0.757)	Model 7	+0.0040 (0.0011–0.0070)
Model 9	Model 8 + Slope-adjusted BMI-SD ≥0.5 kg/m^2 (5th and 6th sextiles*)	5156	0.757 (0.755–0.759)	Model 8	+0.0014 (-0.0014–0.0043)

C-statistics were calculated at two years using time-dependent areas under the receiver operating characteristic curve for competing risk analysis, with standard errors obtained using the DeLong method.

* The 1st–2nd sextile [1st tertile] and 6th sextile of annual BMI change and the 5th–6th sextile [3rd tertile] of slope-adjusted BMI-SD were significantly associated with dementia risk as shown in **Figure 2**.

AIC, Akaike Information Criterion; BMI, body mass index; CI, confidence interval; HbA1c, hemoglobin A1c; LDL, low-density lipoprotein; SD, standard deviation.

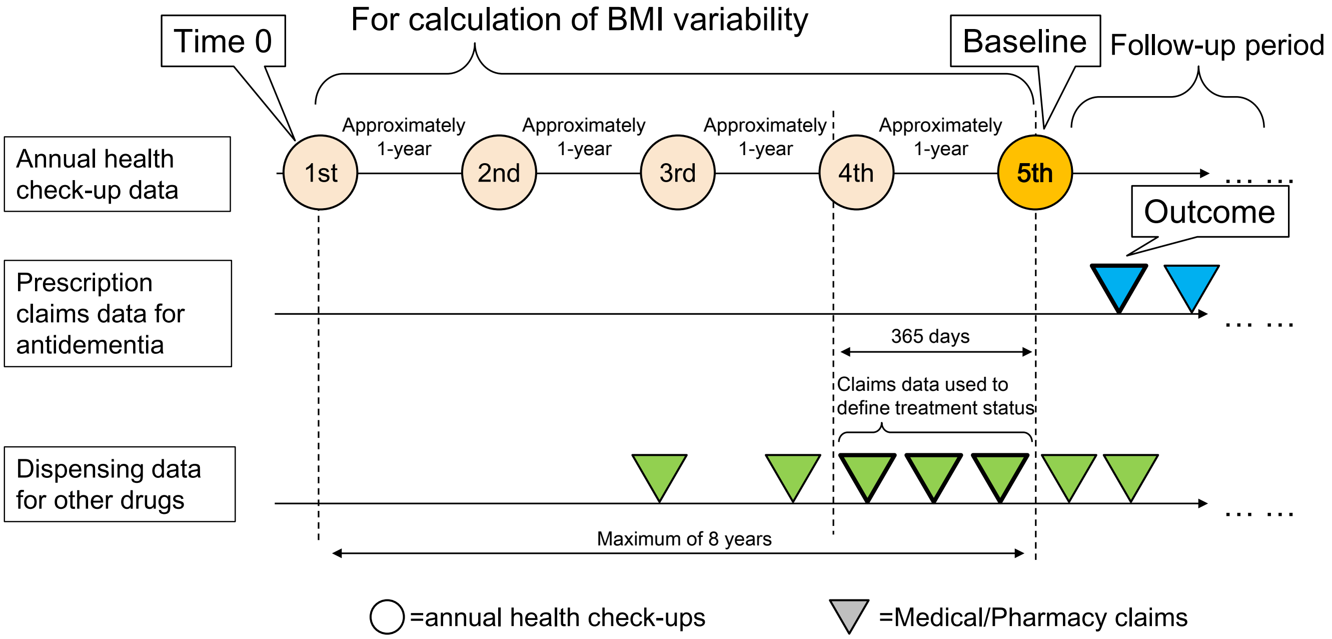

Figure S1. Scheme of Study Design

The 5th health check-up served as the baseline, with anti-dementia treatment status defined using prescription claims data from within 365 days of the baseline. The maximum period to observe visit-to-visit BMI variability was eight years.

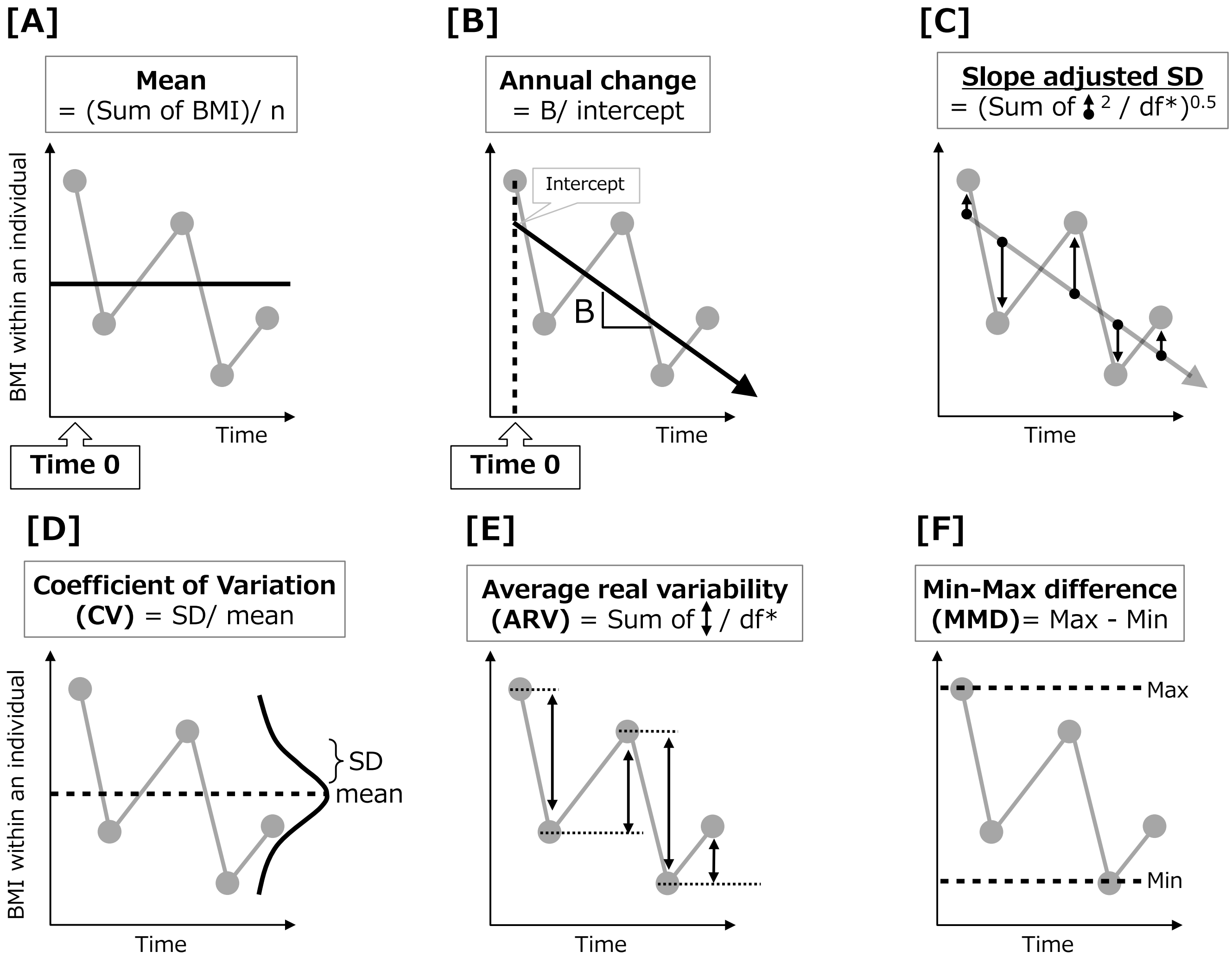

Figure S2. Illustration of Variability Measurement Methods

Schematic representation of different approaches to quantify variability over time.

[A] **Mean, unit**: calculated as the sum of values divided by the number of measurements.

[B] **Annual change, %**:
calculated as the slope from the linear regression divided by the intercept at baseline (Time 0).

[C] **Slope-adjusted SD, unit**:
calculated as the square root of the sum of squared deviations from the fitted trend line divided by the degrees of freedom. *The degree of freedom (df) was set at 2 (accounting for the regression coefficient and intercept)

[D] **Coefficient of variation (CV), %**:
ratio of standard deviation to mean BMI across all time points.

[E] **Average real variability (ARV), unit**:
sum of absolute differences between consecutive BMI measurements divided by degrees of freedom.

[F] **Min-max difference (MMD), unit**:
absolute difference between the highest and lowest BMI values during follow-up.

BMI, body mass index

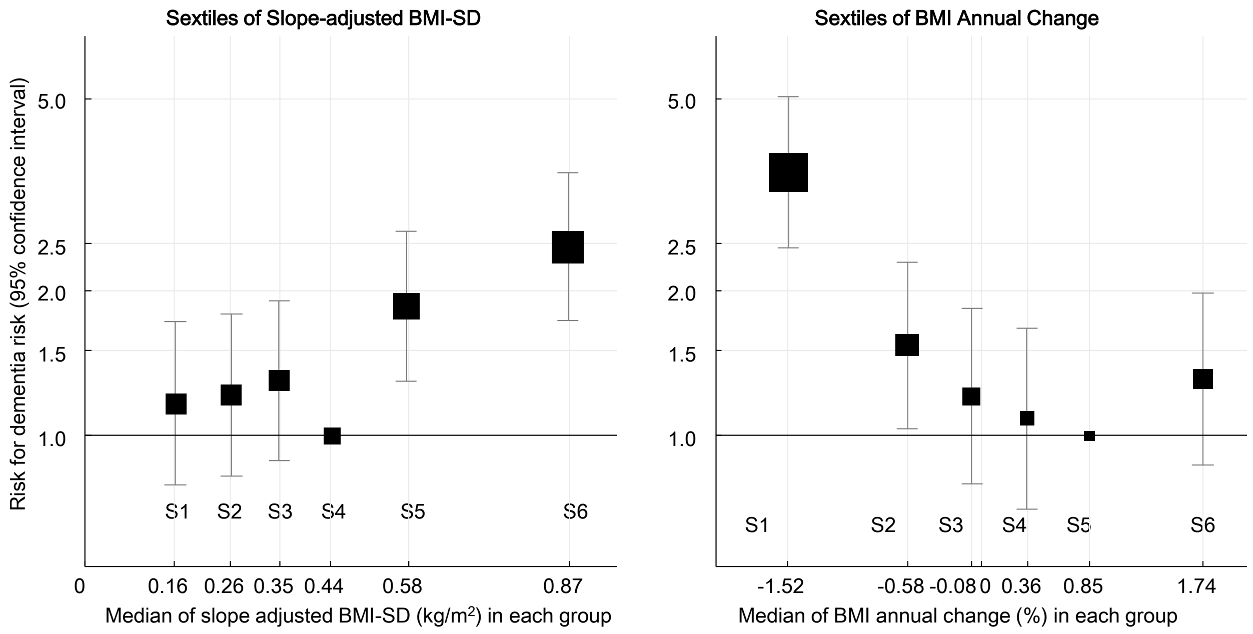

Figure S3. Association Between BMI Trajectories and Dementia Risk in Females

Hazard ratios for anti-dementia drug initiation across sextiles for annual BMI change from baseline (left panel) and slope-adjusted BMI-SD (right panel) among female participants. Both BMI trends and variability measures were simultaneously included in the models with covariates to assess independent associations. The models were adjusted for age, smoking status, alcohol consumption, proteinuria, systolic blood pressure, HbA1c and LDL cholesterol levels, medication use, and BMI at baseline. The lowest risk group was used as the reference category.

BMI, body mass index; HbA1c, hemoglobin A1c; LDL, low-density lipoprotein; SD, standard deviation

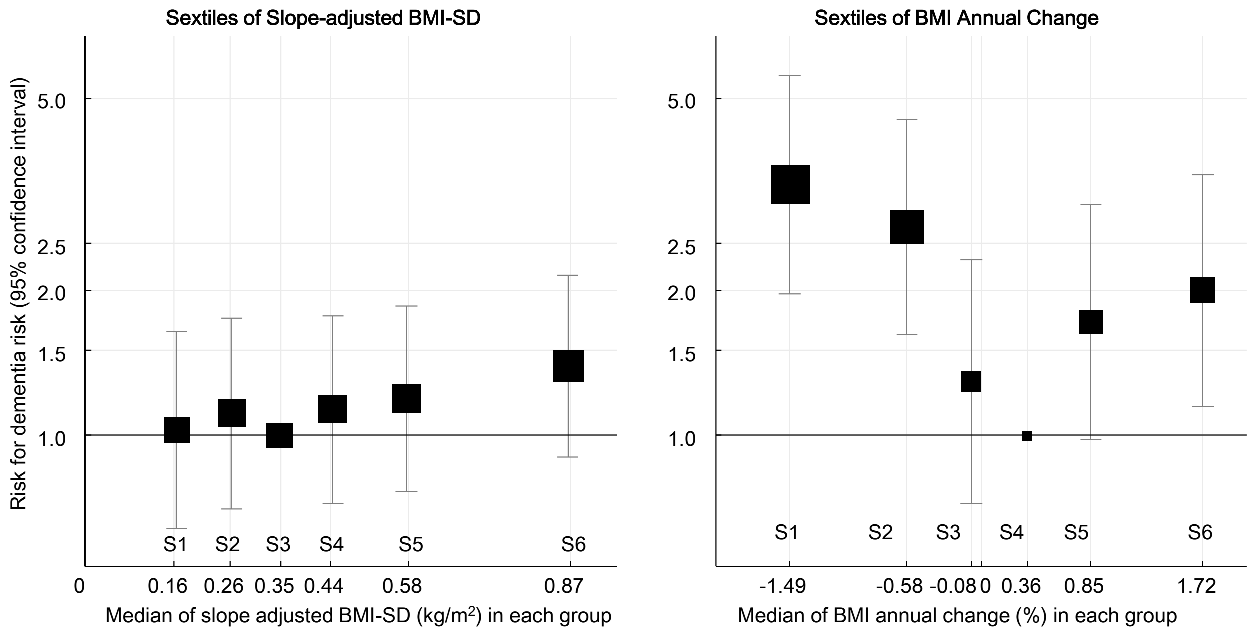

Figure S4. Association Between BMI Trajectories and Dementia Risk in Males

Hazard ratios for anti-dementia drug initiation across sexes for annual BMI change from baseline (left panel) and slope-adjusted BMI-SD (right panel) among male participants. Both BMI trends and variability measures were simultaneously included in the models with covariates to assess independent associations. The models were adjusted for age, smoking status, alcohol consumption, proteinuria, systolic blood pressure, HbA1c and LDL cholesterol levels, medication use, and BMI at baseline. The lowest risk group was used as the reference category.

BMI, body mass index; HbA1c, hemoglobin A1c; LDL, low-density lipoprotein; SD, standard deviation
